# Essential Oils from Indigenous Iranian Plants: A Natural Weapon vs. Multidrug-Resistant *Escherichia coli*

**DOI:** 10.3390/microorganisms10010109

**Published:** 2022-01-05

**Authors:** Mohammadreza Pajohi Alamoti, Behnaz Bazargani-Gilani, Razzagh Mahmoudi, Anna Reale, Babak Pakbin, Tiziana Di Renzo, Ata Kaboudari

**Affiliations:** 1 Department of Food Hygiene and Quality Control, Bu-Ali Sina University, Hamedan P.O. Box 6517658978, Iran; Pajohi@gmail.com (M.P.A.); b.bazargani@basu.ac.ir (B.B.-G.); 2 Medical Microbiology Research Center, Qazvin University of Medical Sciences, Qazvin P.O. Box 34185-754, Iran; b.pakbin@ut.ac.ir; 3 Institute of Food Science, National Research Council (ISA-CNR), Via Roma 64, 83100 Avellino, Italy; anna.reale@isa.cnr.it (A.R.); tiziana.direnzo@isa.cnr.it (T.D.R.); 4 Department of Food Hygiene and Quality Control, Urmia University, Urmia P.O. Box 1177, Iran; a.kaboudari@urmia.ac.ir

**Keywords:** antibiotic susceptibility, pathogen, *Zataria multiflora*, essential oils, dairy products

## Abstract

Aim of this study was to investigate the antimicrobial properties of herbal plant essential oils (EOs) from selected Iranian plant species such as *Ferulago angulata*, *Zataria multiflora*, *Cuminum cyminum*, and *Mentha longifolia* against antibiotic-resistant *Escherichia coli* (*E. coli*) strains. For this purpose, the *Escherichia coli* strains, isolated from raw cow’s milk and local dairy products (yogurt, cream, whey, cheese, and confectionery products) collected from different areas of Hamedan province, Iran, were investigated for their resistance to antibiotics (i.e., streptomycin, tetracycline, gentamicin, chloramphenicol, ciprofloxacin, and cefixime). Thus, the *E. coli* strains were tested for their susceptibility to the above-mentioned essential oils. Regarding antibiotics, the *E. coli* strains were highly sensitive to ciprofloxacin. In relation to essential oils, the most effective antibacterial activity was observed with *Zataria multiflora*; also, the bacteria were semi-sensitive to *Cuminum cyminum* and *Mentha longifolia* essential oils. All strains were resistant to *Ferulago angulata* essential oil. According to the results, the essential oil of *Zataria multiflora* can be considered as a practical and alternative antibacterial strategy to inhibit the growth of multidrug-resistant *E. coli* of dairy origin.

## 1. Introduction

*Escherichia coli* (*E. coli*) is one of the most important microorganisms causing infections of the digestive and urinary tracts in humans and animals. The main way of transmission of this pathogen is through the fecal–oral chain; however, the handling of food during processing is considered risky because it can allow the transfer of this pathogen [1]. Furthermore, *E. coli* is one of the most important and leading causes of mastitis in cattle, from which the pathogen can easily be transmitted to consumers through milk and dairy products [2]. In this regard, due to the excessive use of antibiotics used to control these diseases, significant resistance to a wide range of antibiotics has developed in several *E. coli* strains [3,4]. For this reason, the presence of the multi-drug pathogens in foods of animal origin such as milk, meat, and poultry has dramatically increased in recent years [5]. So, the consumption of high-risk foods including raw milk and artisanal (traditional) products such as cheese, yoghurt, whey, cream, creamy desserts, and roulette cakes can play an important role in the transmission of these multi-drug resistant pathogens. In the last decades, many strategies have been undertaken to fight multidrug-resistant bacterial infections, including phage therapy, new vaccines, and new peptides, to name a few [6]. In addition, many studies are focusing on finding new and effective antimicrobial agents such as essential oils (EOs), aromatic oily liquids obtained by fermentation, extraction or steam distillation of plant material (flowers, buds, seeds, leaves, twigs, bark, herbs, wood, fruits, and roots) [7,8,9].

Furthermore, EOs are generally accepted by consumers thanks to their high volatility and biodegradable nature [10,11].

Many plant oils or extracts have been reported to have antimicrobial properties and this is attributed to their ability to synthesize aromatic substances, most of which are phenols or oxygen-substituted derivatives [12].

Phenolic compounds in essential oils provide the antimicrobial characteristics through different action at the microbial cellular level such as modification of cell membrane permeability; coagulation of cell contents; changes in various intracellular functions induced by hydrogen binding of phenolic compounds to enzymes or by modification of cell wall rigidity with loss of integrity due to different interactions with the cell membrane [13].

In recent years, many researches have increasingly demonstrated the antimicrobial effect of different essential oils (such as cilantro, coriander, oregano, cinnamon, rosemary, sage, clove, thyme, etc.) against pathogenic bacteria and food spoilage agents [14,15,16,17] and have proposed new solutions to deliver these compounds [18,19]. Wang et al. (2020) showed that ginger essential oil, where zingiberene and α-curcumene were identified as the main chemical constituents, has excellent antibacterial activity against *E. coli* and *S. aureus* [20]. Al-Nabulsi et al., (2020) demonstrated that the EOs of cinnamon and thyme showed the strongest inhibition against *E. coli* while sage, radish seeds, black seeds, mustard, and pomegranate seeds showed no inhibitory effect against *E. coli* O157:H7 [21]. Silva et al. (2020) in a meta-analysis study highlighted that lemon balm, sage, shallot, and anise EOs had the best inhibitory results against the pathogen *E. coli* [22]. 

The aim of the present research is to investigate the antimicrobial activity of EOs of indigenous plants growing wild and abundantly in Southwest Asia (Iran, Afghanistan, and Pakistan) such as *Mentha longifolia*, *Cuminum cyminum*, *Zataria multiflora*, and *Ferulago angulata* against *E. coli*. These plants were selected on the basis of their production and consumption in Iran, where they are widely used in food and pharmaceutical industries [23,24]. The main chemical composition of the EOs was determined by Gas Chromatography-Mass Spectrometry (GC/MS) technique. The *E. coli* strains used in this study were isolated from raw cow’s milk and local dairy products (yogurt, cream, whey, cheese, and confectionery products) collected from different areas of Hamedan province, Iran. The isolates, after appropriate identification, were tested for their susceptibility to antibiotics, such as Cefixime (CF), Chloramphenicol (C), Ciprofloxacin (CP), Gentamicin (GM), Streptomycin (S), and Tetracycline (TE).

## 2. Materials and Methods

### 2.1. Sampling 

A total of 180 samples of raw cow’s milk and local dairy products (yoghurt, cream, whey, cheese, and confectionery products) were collected from different areas of Hamedan province, Iran (see Table 1). The samples were aseptically transferred under cold and sterile conditions to the food hygiene laboratory of Bu-Ali Sina University, Hamedan, for the microbiological and chemical analysis. Isolation and identification of presumptive *E. coli* were carried out using conventional cultural methods and biochemical analysis. Presumptive *E. coli* isolates were subsequently genetically identified by Polymerase Chain Reaction (PCR) techniques, as described below. Then, the susceptibility of *E. coli* strains to essential oils (EOs) of herbal plants and to antibiotics was assessed.

### 2.2. Microbiological Analysis

For *E. coli* isolation, 10 g (or mL) of each homogenized sample was suspended into 90 mLof Trypticase Soy Broth (TSB supplemented with 20 mg/L Novobiocin) and incubated at 37 °C for 18–24 h. The enriched cultures were streaked on McConkey lactose agar and incubated at 37 °C for 18–24 h. Pink colonies were selected as presumptive *E. coli* colonies and streaked on Nutrient agar slant. Then the isolates were subjected to conventional assays including Gram staining, growth on Eosin Methylene Blue agar, and Triple Sugar Iron agar (TSI agar) (Merck, Darmstadt, Germany) and biochemical tests such as Indole, Methyl red, and Voges-Proskauerand Citrate utilization tests (IMViC) and Catalase test. All media and supplements, when not differently specified, were provided by Merck, Darmstadt, Germany. Presumptive *E. coli* isolates *(n* = 35) were assayed by genetic identification as described below.

### 2.3. DNA Extraction 

DNA was extracted from the isolates using the boiling method [25]. Briefly, a 2-mL overnight bacterial culture was centrifuged for 5 min at 3600 rpm and the pellet was re-suspended in 200 μL of sterile distilled water. The bacterial suspension was boiled at 100 °C for 10 min in a water bath and cooled at refrigerator temperature. Once again, the suspension was centrifuged at 12,000 rpm for 2 min and the supernatant was used as the template DNA in polymerase chain reaction (PCR) [26].

### 2.4. PCR

Amplifications were performed in a gradient thermocycler (Applied Biosystems™ Veriti™ Thermal Cycler, Thermo Fisher Scientific, Waltham, MA, USA) using the primers Eco 2083 F (5′-GCTTGACACTGAACATTGAG-3′) and Eco 2745 R (5′-GCACTTATCTCTTCCGCATT-3′), specific for *E. coli* and targeted to the 23S rRNA gene [27]. The PCR reaction was carried out in a final volume of 25 μL, containing 4–5 μL of template DNA, 2.5 μL of 10× PCR buffer, 0.75 μL of 50 mM MgCl2, 0.5 μL of 10 mM deoxyribonucleoside triphosphates (dNTP), 0.25 μL of 5 U/μL of Taq DNA polymerase (Sinaclon, Tehran, Iran), and 10 pmol of each primer. The PCR amplification was performed in 35 cycles using the following conditions: initial denaturation at 94 °C for 5 min; denaturation at 94 °C for 1 min, annealing at 57 °C for 1 min, extension at 72 °C for 2 min; and final extension at 72 °C for 7 min. *E. coli* strain ATCC 25922 and distilled water were used as positive and negative standard controls, respectively. PCR products were analyzed by electrophoresis on 2% agarose gel (Sinaclon, Tehran, Iran) containing ethidium bromide (0.5 μg/mL) under ultraviolet (UV) light. PCR gels were digitally captured by GEL DOC XR System (Bio-Rad, Hercules, CA, USA).

### 2.5. Antibiotic Susceptibility Assay 

According to the guidelines of CLSI (Clinical and Laboratory Standards Institute) standards (2015) antimicrobial susceptibility tests were done on Mueller–Hinton agar (Merck, Darmstadt, Germany) using Kirby–Bauer disk diffusion method [28].

The antibiotic discs (padtanTeb. Co., Tehran, Iran) tested were: Streptomycin (10 μg) (S), Tetracycline (30 μg) (TE), Gentamycin (10 μg) (GM), Chloramphenicol (30 μg) (C), Ciprofloxacin (5 μg) (CP), and Cefixime (5 μg) (CFM). The diameter of each inhibition zone was measured in millimeters after incubation at 37 °C for 20 h.

### 2.6. Plant Material 

Dried plants of *Mentha longifolia* L., *Zataria multiflora* Boiss., *Ferulago angulata* (Schlecht.) Boiss, and *Cuminum cyminum* L. were purchased from local markets of Tehran, Iran; then identified in the Institute of Medicinal Plants, Tehran, Iran and in the Faculty of Agriculture, Bu-Ali Sina University, Hamedan, Iran. The plants were identified by Dr. Azizi inthe Department of Horticulture, Bu-Ali Sina University, Hamedan, Iran. *Mentha longifolia* was collected near the city of Urmia, Iran, in August 2013. It was identified by Dr. Shahrokh Kazempour, from the Department of Plant Biology of the Tarbiat Modares University, Tehran, Iran. A voucher specimen (number 3571-B) of this plant has been deposited in the Herbarium of the Institute. *Zataria multiflora* Boiss., a voucher specimen has been deposited at the Herbarium of the Faculty of Pharmacy, Kerman, University of Medical Sciences, Kerman, Iran (KF1375). *Ferulago angulata* (Schlecht.) Boiss., a voucher specimen (Number 3054) has been deposited at Herbarium of Agriculture and Natural Resources Research Center of Kermanshah. *Cuminum cyminum* L., the plant was authenticated and voucher specimen (C-1456) has been deposited in the Herbarium of the Department of Pharmacognosy, Faculty of Pharmacy, Shaheed Beheshti University of Medical Sciences, Tehran.

### 2.7. Extraction and Analysis of Essential Oil 

The hydro-distillation method using Clevenger-type apparatus was applied to extract the EO from the herbal plants. The main chemical composition of the EOs was determined by gas chromatography (GC) method (Agilent 6890, New York, NY, USA) equipped with a flame ionization detector (FID) and coupled with a Mass Spectrophotometer (MS) (Agilent 5973). A gas chromatography/mass spectrometry (GC/MS) system and the capillary column HP-5 (30 mm × 0.25 mm ID) with a layer thickness of 0.25 μm were used for chemical analysis of EOs. Helium was used as a carrier gas. The sample injection volume was 1 μL and methanol was used as solvent. The identification of the compounds was done using Wiley Library. Quantification was done by the external standard method using the calibration curves generated by performing GC analysis of the representative compounds. Data processing was performed using Chemstation software. 

### 2.8. In Vitro Detection of EOs Antimicrobial Activity 

The antimicrobial activity of *Mentha longifolia* L., *Zataria multiflora* Boiss., *Ferulago angulata* (Schlecht.) Boiss and *Cuminum cyminum* extracts was assessed against 19 *E. coli* strains by agar well diffusion assay [29]. Then, 1 mL (inoculum size of 106 CFU/mL) of each bacterial suspension was inoculated into 20 mL of proper soft media (0.7% agar), gently mixed and poured into Petri plates. For the determination of the antimicrobial activity of EOs, sterile paper discs (diameter 6 mm) were impregnated with 10 µL of each EO and located on the inoculated plates. After incubation at 37 °C for 24 h, inhibition halos (mm) were accurately measured. Thereafter, 3 levels of inhibition were established: strong (>25 mm), moderate (15–25 mm), and low (<15 mm). The presence of growth around the well was considered as absence of inhibition. All experiments were performed in triplicate. Data were reported as the mean value and standard deviation. 

### 2.9. Statistical Analysis 

All statistical analyses were carried out with SPSS software ver. 20.0.0.1 (Chicago, IL, USA). Susceptibility was calculated as percentages with 95% confidence intervals and a *p*-value of data analysis. The relationships between categorical variables were examined using the Chi-square test with significance set at a *p* ≤ 0.05. 

## 3. Results

### 3.1. Isolation and Identification of E. coli Strains

Among the 180 samples analyzed, there were 35 presumptive *E. coli* detected based on morphological and cultural characteristics and also biochemical tests (Table 1); in detail, 13 colonies from milk, five from cheese, five from cream and confectionary products, seven from whey, and five from yoghurt.

**Figure 1:**
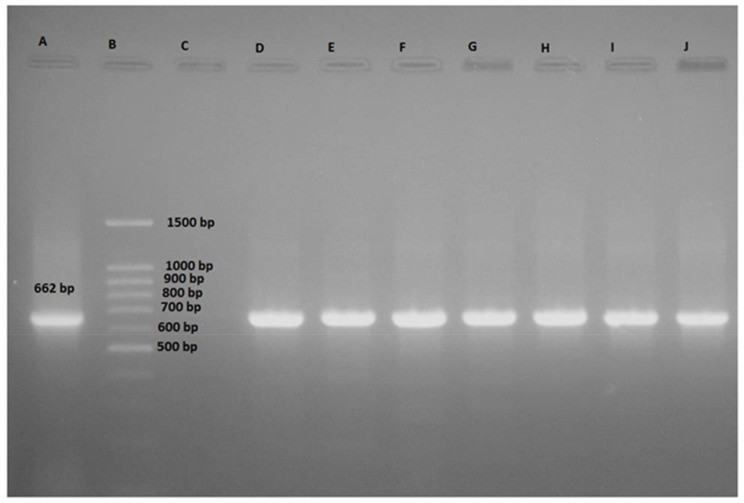
Gel electrophoresis of PCR products amplified with Eco 2083 F and Eco 2745 R primers with 662 bp. Lane A: positive control, PCR product amplified with DNA from *E. coli* strain ATCC 25,922; Lane B: 100-bp DNA ladder; Lane C: no template; Lane D–J: show the PCR products amplified with DNA from *Escherichia coli* strain RE11, RE12, RE13, RE14, RE15, RE16, and RE17, respectively.

The 35 bacterial isolates recovered were subjected to molecular analysis by DNA extraction followed by partial sequencing of their 23S rDNA. Results of sequence analysis (Table 1, Figure 1) showed that only 19 isolates belonged to *E. coli* species. Among them, seven were recovered from milk, three from cheese, two from cream and confectionary products, three from whey, and four from yoghurt.

### 3.2. Antibiotic Susceptibility of E. coli Strains

The antibiotic susceptibility of *E. coli* strains is shown in Table 2. All strains were susceptible to ciprofloxacin (inhibition halo > 36 mm in diameter) and were moderately susceptible to tetracycline (TE), gentamicin (GM), streptomycin (S), cefixime (C), and chloramphenicol (CFM). In particular, GM, CFM, C, and TE produced an inhibition halo of approximately 24 mm in diameter (medium value) while streptomycin produced an inhibition halo that was 18.2 mm in diameter (mean value).

Table 3 shows the compositions of essential oils of selected Iranian plant species analyzed by Gas Chromatography-Mass Spectrometry technique.A total of 46 volatile organic compounds were identified in the different plant extracts. Results indicated that *Zataria multiflora* EO was mainly represented by oxygenated monoterpenes (OMs) (76.10%) and hydrocarbon monoterpenes (MTs) (13.07%) and a low percentage of sesquiterpenes (STs) (2.73%). Carvacrol (71.12%), and eucalyptol (3.37%) were the main components of OMs, while α-terpinene (7.34%) and α-pinene (4.26%) were the main components of MTs. The essential oil of *Ferulago angulata* was mainly represented by hydrocarbon monoterpenes (95.64%) and a very low percentage of oxygenated monoterpenes (2.01%), sesquiterpenes (2.68%), and other minor compounds (0.93%). Specifically, the main compounds were β-ocimene (40.02%), α-phellandrene (14.47%), β–phellandrene (14.43%), terpinolene (6.96%), α-pinene (4.61%), p- cymene (4.12%), and β-cymene (2.62%). The main components of *Cuminum cyminum* EO were represented by oxygenated monoterpenes (59.88%), hydrocarbon monoterpenes (32.77%), sesquiterpenes (0.37%), and other minor compounds (4.45%). In detail, cuminaldehyde (29.00%), α-terpinene-7-al (20.7%), ɣ-terpinene (12.94%), ɣ-terpinene-7-al, and p-cymene (8.55%) were the main components. The essential oil of *Mentha longifolia* L. contained mainly oxygenated monoterpenes (67.36%) represented mainly by pulegone (31.54%) and 1,8-cineole (15.89%). Hydrocarbon monoterpenes (6.52%) and other compounds (18.42%) were represented by menthofuran (11.18%). 

**Figure 2:**
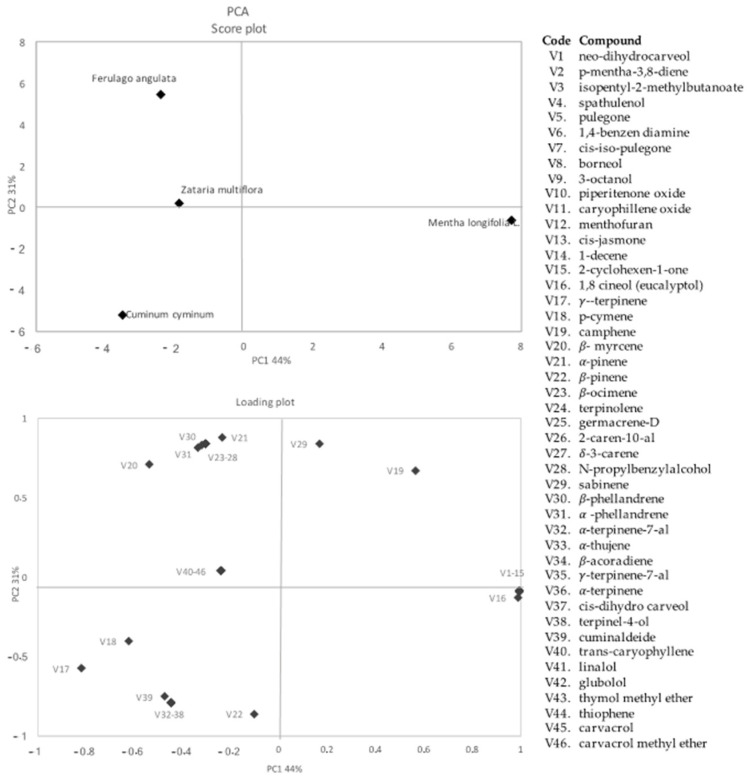
Score plot and loading plot of first and second principal components after principal component analysis based on volatile components that differentiated the essential oil of *Ferulago angulate*, *Zataria multiflora*, *Cuminum cyminum*, and *Mentha longifolia*. VOCs used in PCA are listed in the legend on the right of the figure.

In order to better understand the differences occurring among the essential oils from the different Iranian plants, a PCA of the 46 recorded volatile compounds was calculated, as shown in Figure 2. The two PCs explained about 75% of the total variance of the data. 

Several substances positively loaded on PC1, including high positive loadings for 1,8-cineole, pulegone, cis-iso-pulegone, p-mentha-3-8, diene, neo-dihydrocarveol, caryophyllene oxide, and menthofuran. Regarding PC2, the main positive contribution was due to αcamphene, sabinene, α-pinene, α-phellandrene, β-phellandrene, α-mircene, and α-ocimene. 

EOs, as determined by the two PCs (factors), were distributed in different zones of the plan. Regarding the scores plot, *Mentha longifolia* EO was located in the right part of the plan and it was totally different from the other EOs; it was characterized by the presence of menthofuran, pulegone, p-mentha-3,8-diene, 1,8-cineol; *Ferulago angulata* showed positive scores on the PC2 and negative score for PC1 and was entirely located in the higher left section of the graph; it was characterized by ocimene, α-mircene, α- and β-phellandrene, whereas *Cuminum cyminum* was located in the lower left section. *Zataria multiflora* essential oil was located in the center of the plan and was characterized for the presence of carvacrol, globulol, thymol linalol, and thiophene (Figure 2). 

### 3.3. Antimicrobial Effects of Essential Oils on Bacterial Isolates

The results of the antimicrobial activity of essential oils are shown in Table 4. Clear inhibition zones around the discs indicated the presence of antimicrobial activity against *E. coli* strains. No halo of inhibition indicated any antibacterial activity. The strains showed different behavior depending on the extract.

*E. coli* strains were more sensitive to *Zataria multiflora* EO than other EOs. In particular, *Zataria multiflora* EO resulted in the largest inhibition halos (between 25.8 and 36.5 mm in diameter) compared to *Mentha longifolia* EO (between 10.5 and 14.7 mm) and *Cuminum cyminum* (11.3–13.5 mm). The susceptibility of *E. coli* strains to EO of *Cuminum cyminum* and *Mentha longifolia* L. were very similar. Only *Ferulago angulata* extract had no antibacterial activity against the tested strains.

## 4. Discussion

In this research, *E. coli* strains were isolated from several samples collected from different areas of Hamedan province, Iran, highlighting those dairy products available on the market still have considerable *E. coli* contamination. *E. coli* isolates were found not only in milk samples (36.8%), but also in different cheeses (15.8%), cream and confectionary products (10.6%), whey (15.8%), and yoghurt (21%) warning of the need for more stringent preventive measures to avoid *E. coli* contamination. As also highlighted by different studies, the prevalenceof hygiene measures and frequency of *E. coli* in food in Iran varies depending on the regions and the type of products, mainly dairy products [30,31]. 

Thus, in this study, a total of 19 *E. coli* strains, were tested against six antibiotics according to CLSI guidelines.

Ciprofloxacin, gentamicin, cefixime, and tetracycline were found to be the most effective antibiotics against these *E. coli* strains. 

Some similarities were found with Rezaei et al. (2019), who found in *E. coli* strains isolated from pastry cream prepared in Hamadan, Iran, the highest resistance to tetracycline, vancomycin, oxacillin (100%), and penicillin (72.34%) and the highest sensitivity to chloramphenicol (78.23%), ciprofloxacin (76.59%), and nalidixic acid (61.70%) [32]. Also, Alizadeh (2018) highlighted that over the years in Iran, antibiotic resistance in Gram-negative bacteria, particularly *E. coli*, has increased significantly. The similarities and the differences observed in the susceptibility pattern may be related to changes in the concentration and frequency of use of these antibiotics in farms. Therefore, it is necessary to consider effective control measures (prophylactic management, hygiene) to reduce the emergence and spread of drug-resistant *E. coli* bacteria in dairy products [33]. 

So, the multi-resistant antibiotic *E. coli* strains, isolated in this study, were tested for their susceptibility to the essential oils from four different Iranian plants. Specifically, the essential oils of *Mentha longifolia* L., *Zataria multiflora*, *Ferulago angulata*, and *Cuminum cyminum* analyzed in this research were mainly characterized by monoterpenes and oxygenated monoterpenes and the profiles of volatile organic compounds agreed with previous studies, although the composition of plant EOs can change depending on several factors such as geographical region, harvest time, extraction method, etc. [34].

*E. coli* strains were found to be differently sensitive to the essential oils extracted from the plants analyzed in this study. In particular, EO of *Zataria multiflora* strongly inhibited all the *E. coli* strains. Our results agree with other authors who have shown that *Zataria multiflora* EO had an inhibitory effect on growth of *E. coli* O157 in white-brined cheese and in traditional Lighvan cheese [35]. 

The strong inhibitory effect of *Z. multiflora* EO on all 19 *E. coli* strains, can be related to the high carvacrol content (71.12%) detected (Table 3; Figure 2), as also highlighted by other authors who reported that carvacrol, thymol, linalool, and p-cymene are responsible for the antimicrobial and antioxidants attributes of *Zataria multiflora* [36,37].

Several studies have shown that the active chemical groups of these compounds, such as hydroxyl groups, react strongly with different vital components of microorganisms leading to the disruption of sensitive molecules such as DNA and metabolic enzymes [38,39,40].

Also, EOs of *Mentha longifolia* and *Cuminum cyminum* showed moderate antimicrobial activity against all 19 *E. coli* strains. The inhibitory effects of *Cuminum* extract on multidrug-resistant *E. coli* O:157 have been demonstrated previously by other authors [41,42]. Iacobellis et al. (2005) showed that the antibacterial activity of *Cuminum cyminum* EO against some gram-negative and positive bacteria is due to the presence of high levels of cuminaldehyde, which agree with the results obtained in this study [43]. 

Recently, Monteiro-Neto et al. (2020) showed that cuminaldehyde was antimicrobial against several strains of *S. aureus* and *E. coli* and that cuminaldehyde may be useful as an adjuvant to ciprofloxacin therapy. As is evident from the analysis of the volatile components (Figure 2), *Cuminum cyminum* essential oil was mainly characterized by cuminaldehyde (29%), together with α-pinene, α-terpinene, and α–terpinene [44].

Regarding *Mentha longifolia* EO, our results are in accordance with previous findings that showed the antimicrobial effect of *Mentha longifolia* EO, consisting mainly of pulegone and piperitone oxide [45,46]. Furthermore, Nikšić et al. (2012) highlighted that the main constituents of the essential oil of *M. longifoliae* were oxygenated monoterpenes, piperitone oxide (63.58%), and 1,8-cineole (12.03%) and that the most important antibacterial activity of essential oil was expressed on Gram negative strains: *Escherichia coli*, *Pseudomonas aeruginosa*, and *Salmonella enterica* [47].

Regarding essential oil of *Ferulago angulata*, no antimicrobial effect has been recorded against all 19 *E. coli* isolates. Similarly, Taran et al. (2011) reported that the essential oil from the aerial parts of *F. angulata* had the higher antibacterial activity against *S. aureus*, but had no significant activity against *Shigella boidii*, *Pseudomonas aeruginosa*, *E. coli*, and *Enterococcus faecalis* [48].

Other researchers demonstrated that gram-positive bacteria, such as *Staphylococcus aureus* and *Streptococcus agalactia*, were more susceptible to *Ferulago angulata* EO than gram-negative ones such as *Escherichia coli* and *Klebsiella pneumonia* [49]. Also, Tabatabaei Yazdi et al. (2014) observed antimicrobial activity of ethanolic and aqueous extracts of *Ferulago angulata* against *Staphlococcus epidermidis*, *Enterobacter aeruginosa* and *Yersinia enterocolitica.* Furthermore, the highest diameter of the inhibition zone growth was for *Staphylococcus epidermidis* and the lowest was measured for *Enterobacter aeruginosa* [50]. Gram-positive bacteria appeared to be more sensitive to *Ferulago angulata* EO because of the thick mucopeptide layer included in the cell wall of gram-positive bacteria which is thinner in the gram-negative ones. As suggested by Alizadeh et al. (2013), lipoprotein and lipopolysaccharide compounds in the cell wall of gram-negative bacteria makes them stronger and resistant to such antibacterial agents [51]. As underlined by Tabatabaei Yazdi et al. (2015) Gram negative bacteria have effective permeability barrier, comprised of the outer membrane, which limits the penetration of amphiphatic compounds and multidrug resistance pumps that extrude toxins across this barrier. It is possible that the apparent ineffectiveness of some plant antimicrobial activity is largely due to this permeability barrier [52]. 

Furthermore, a previous study highlighted that factors determining the activity of essential oils are the composition and functional groups present in active components [53]. Recently, Guimarães et al. (2019) stated that oxygenated functional groups in terpenic compounds exhibited better antimicrobial activity than hydrocarbons. This statement agrees with the present work, since, the essential oils that exerted the highest antimicrobial activity against *E. coli* strains were *Zataria multiflora*, *Mentha longifolia*, and *Cuminum cyminum* EO, that were characterized by the highest percentage of oxygenated monoterpenes (76.1%, 67.4%, and 59.9%, respectively), with respect to *Ferulago angulata* (2.01%) [54].

## 5. Conclusions

According to the finding of the present study, the *E. coli* strains isolated from dairy products were highly susceptible to ciprofloxacin. The EOs of *Zataria multiflora*, *Mentha longifolia* L., and *Cuminum cyminum* strongly inhibited the multi-resistant *E. coli* strains. The most effective antibacterial activity was observed for *Zataria multiflora* while all strains were resistant to *Ferulago angulata* essential oil. Moreover, the abundance, low cost, easy access, and indigenous nature of the herbal plants in Iran, as well as conceptual acceptance by consumers could lead to the introduction of these EOs into the food industry by improving food safety.

## Figures and Tables

**Table 1:** Number of samples (milk and dairy products) having *E. coli* isolates.

Sources	Area	Number of Samples	N. of Presumptive *E. coli* Colonies	N. of Positive *E. coli* by Genetic Identification	
				No.	%
**Raw milk**	Hamedan province	40	13	7	36.8
**Cheese**	Hamedan province	35	5	3	15.8
**Cream and** **confectionary products**	Hamedan province	35	5	2	10.6
**Whey**	Hamedan province	35	7	3	15.8
**Yoghurt**	Hamedan province	35	5	4	21.0
**Total**		180	35	19	100

**Table 2:** Antibiotic Susceptibility of *E. coli* strains (inhibition halo in diameter, mm).

	Source of Isolation (Number of Strains)					
Antibiotic	Raw Milk (*n* = 7)	Yogurt (*n* = 4)	Cream (*n* = 1)	Whey (*n* = 3)	Cheese (*n* = 3)	Confectionary Products (*n* = 1)
GM	23.5 ± 0.4 ^Aa^	24.1 ± 0.4 ^Aa^	24.5 ± 0.3 ^Aa^	23.9 ± 0.1 ^Aa^	22.5 ± 0.2 ^Aa^	24.8 ± 0.4 ^Aa^
S	15.6 ± 0.2 ^Ba^	18.5 ± 0.6 ^Bb^	19.7 ± 0.3 ^Bb^	17.4 ± 0.4 ^Ba^	14.2 ± 0.4 ^Ba^	23.6 ± 0.1 ^Ac^
CFM	22.3 ± 0.5 ^Aa^	24.3 ± 0.1 ^Aa^	25.4 ± 0.2 ^Ab^	23.1 ± 0.1 ^Aa^	21.4 ± 0.1 ^Aa^	25.2 ± 0.3 ^Ab^
C	25.4 ± 0.5 ^Aa^	25.5 ± 0.4 ^Aa^	25.6 ± 0.1 ^Aa^	26.2 ± 0.3 ^Aa^	25.4 ± 0.3 ^Aa^	25.5 ± 0.1 ^Aa^
CP	40.9 ± 0.1 ^Ca^	38.8 ± 0.5 ^Ca^	37.1 ± 0.2 ^Cb^	39.5 ± 0.1 ^Ca^	40.5 ± 0.8 ^Ca^	36.8 ± 0.2 ^Bb^
TE	24.2 ± 0.7 ^Aa^	24.9 ± 0.9 ^Aa^	24.9 ± 0.5 ^Aa^	23.8 ± 0.7 ^Aa^	22.7 ± 0.3 ^Aa^	25.3 ± 0.9 ^Aa^

**Note:** The diameters of the inhibition zones were measured in millimeters. Streptomycin (S), Tetracycline (TE), Gentamycin (GM), Chloramphenicol (C), Ciprofloxacin (CP), and Cefixime (CFM). Within each column, the uppercase letters (A–C) indicate significant differences (*p* < 0.05) in inhibition halo diameter within the different antibiotic. Within each row, the lowercase (a–c) lower case letters indicate significant differences (*p* < 0.05) in inhibition halo diameter within the different food analyzed.

**Table 3:** Composition of EOs of *Zataria multiflora*, *Ferulago angulata*, *Cuminum cyminum*, and *Mentha longifolia* L. identified by GC/MS.

	Compounds	RT (min)	*Z. multiflora*	*F. angulata*	*C. cyminum*	*M. longifolia* L.
**Hydrocarbon monoterpen (MT)**	β-pinene	4.65	0.43	0.58	7.72	3.07
	α-phellandrene	5.58	nd	14.47	0.79	nd
	β-phellandrene	6.44	nd	14.43	0.34	nd
	α-thujene	7.01	nd	nd	0.34	nd
	α-pinene	7.22	4.26	4.61	0.68	1.86
	camphene	7.76	nd	0.60	nd	0.57
	δ-3-carene	7.78	nd	1.74	nd	nd
	sabinene	9.03	nd	1.11	nd	0.52
	β-myrcene	9.27	0.85	2.62	1.10	0.50
	α-terpinene	10.27	nd	nd	0.31	nd
	p-cymene	10.39	nd	4.12	8.55	nd
	terpinolene	10.53	nd	6.96	nd	nd
	β-ocimene	11.09	nd	40.02	nd	nd
	thiophene	12.01	0.19	nd	nd	nd
	γ-terpinene	12.04	7.34	4.38	12.94	nd
	**Total**		13.07	95.64	32.77	6.52
**Oxygenated monoterpen (OM)**	cis-iso-pulegone	6.48	nd	nd	nd	9.74
	pulegone	7.58	nd	nd	nd	31.54
	p-mentha-3,8-diene	8.22	nd	nd	nd	7.06
	1,8-cineol (eucalyptol)	10.68	3.37	nd	0.84	15.89
	γ-terpinen-7-al	12.04	nd	nd	8.91	nd
	linalol	13.69	0.68	nd	nd	nd
	borneol	16.36	nd	nd	nd	1.01
	terpinel-4-ol	16.96	nd	nd	0.43	nd
	α-terpinen-7-al	17.45	nd	nd	20.70	nd
	cuminaldeide	18.9	nd	1.67	29.00	nd
	carvacrol methyl ether	20.34	0.46	nd	nd	nd
	neo-dihydrocarveol	21.18	nd	nd	nd	1.78
	thymol methyl ether	21.90	0.47	nd	nd	nd
	carvacrol	22.22	71.12	nd	nd	nd
	piperitenone oxide	31.22	nd	nd	nd	0.34
	cis-jasmone	nd	nd	nd	nd	0.40
	2-caren-10-al	nd	nd	0.34	nd	nd
	**Total**		76.10	2.01	59.88	67.76
**Sesquiterpene (ST)**	germacrene-D	16.19	nd	2.68	nd	nd
	trans-caryophyllene	28.27	0.41	nd	nd	nd
	globulol	32.53	2.32	nd	nd	nd
	spathulenol	33.12	nd	nd	nd	0.52
	caryophyllene oxide	33.29	nd	nd	nd	1.60
	β-acoradiene	nd	nd	nd	0.37	nd
	**Total**		2.73	2.68	0.37	2.12
**Others**	1,4-benzen diamine	2.85	nd	nd	nd	0.35
	1-decene	7.15	nd	nd	nd	1.58
	n-propyl benzyl alcohol	10.49	nd	0.93	nd	nd
	isopentyl-2-methyllbutanoate	20.90	nd	nd	nd	0.91
	menthofuran	27.42	nd	nd	nd	11.18
	3-octanol	31.05	nd	nd	nd	0.60
	2-cyclohexen-1-one	32.04	nd	nd	nd	3.80
	cis-dihydro carveol	35.15	nd	nd	4.45	nd
	**Total**		0.00	0.93	4.45	18.42

nd = not determined.

**Table 4:** Antimicrobial activity of EOs against 19 strains of *E. coli* (inhibition zone in diameter, mm *).

N.	Code	Strain	Source of Isolation	*Zataria* *multiflora*	*Mentha* *longifolia* L.	*Cuminum cyminum*	*Ferulago* *angulata*
**1**	***RE11***	***E. coli***	**Raw Milk**	32.4 ± 0.7 ^a^	12.4 ± 0.9 ^a^	13.5 ± 0.9 ^a^	R
**2**	***RE12***	***E. coli***	**Raw Milk**	31.4 ± 0.8 ^ab^	13.4 ± 0.8 ^ac^	13.5 ± 0.8 ^a^	R
**3**	***RE13***	***E. coli***	**Raw Milk**	30.5 ± 0.9 ^b^	10.5 ± 0.7 ^b^	12.5 ± 0.7 ^a^	R
**4**	***RE14***	***E. coli***	**Raw Milk**	31.3 ± 0.8 ^a^	12.5 ± 0.9 ^a^	13.5 ± 0.8 ^a^	R
**5**	***RE15***	***E. coli***	**Raw Milk**	32.4 ± 0.7 ^a^	11.5 ± 0.7 ^ab^	13.5 ± 0.6 ^a^	R
**6**	***RE16***	***E. coli***	**Raw Milk**	32.3 ± 0.8 ^a^	12.3 ± 0.9 ^a^	13.5 ± 0.8 ^a^	R
**7**	***RE17***	***E. coli***	**Raw Milk**	31.4 ± 0.8 ^a^	12.3 ± 0.9 ^a^	11.5 ± 0.8 ^b^	R
**8**	***YE21***	***E. coli***	**Yogurt**	34.5 ± 0.7 ^c^	13.1 ± 0.8 ^ac^	12.9 ± 0.5 ^b^	R
**9**	***YE22***	***E. coli***	**Yogurt**	33.5 ± 0.5 ^ac^	13.2 ± 0.7 ^a^	11.9 ± 0.5 ^b^	R
**10**	***YE23***	***E. coli***	**Yogurt**	35.3 ± 0.5 ^c^	13.1 ± 0.8 ^ac^	12.8 ± 0.5 ^b^	R
**11**	***YE24***	***E. coli***	**Yogurt**	34.4 ± 0.7 ^c^	13.2 ± 0.7 ^ac^	12.7 ± 0.5 ^b^	R
**12**	***CE31***	***E. coli***	**Cream**	35.3 ± 0.4 ^c^	12.9 ± 0.5 ^a^	12.1 ± 0.3 ^b^	R
**13**	***CE32***	***E. coli***	**Confectionary Products**	35.5 ± 0.9 ^c^	11.5 ± 0.2 ^b^	12.5 ± 0.4 ^b^	R
**14**	***WE41***	***E. coli***	**Whey**	30.9 ± 0.7 ^b^	14.7 ± 0.8 ^c^	12.3 ± 0.7 ^b^	R
**15**	***WE42***	***E. coli***	**Whey**	31.8 ± 0.8 ^a^	14.6 ± 0.7 ^c^	12.4 ± 0.8 ^b^	R
**16**	***WE43***	***E. coli***	**Whey**	30.8 ± 0.8 ^a^	13.7 ± 0.6 ^ac^	11.3 ± 0.7 ^b^	R
**17**	***CHE51***	***E. coli***	**Cheese**	36.5 ± 0.2 ^a^	13.5 ± 0.1 ^a^	12.5 ± 0.8 ^b^	R
**18**	***CHE52***	***E. coli***	**Cheese**	25.8 ± 0.2 ^d^	11.2 ± 0.6 ^b^	12.2 ± 0.1 ^b^	R
**19**	***CHE53***	***E. coli***	**Cheese**	30.6 ± 0.4 ^b^	12.2 ± 0.4 ^a^	12.2 ± 0.5 ^b^	R

* The lower case letters in each column indicate a statistically significant difference (*p* < 0.05). R = resistant—absence of inhibition.
